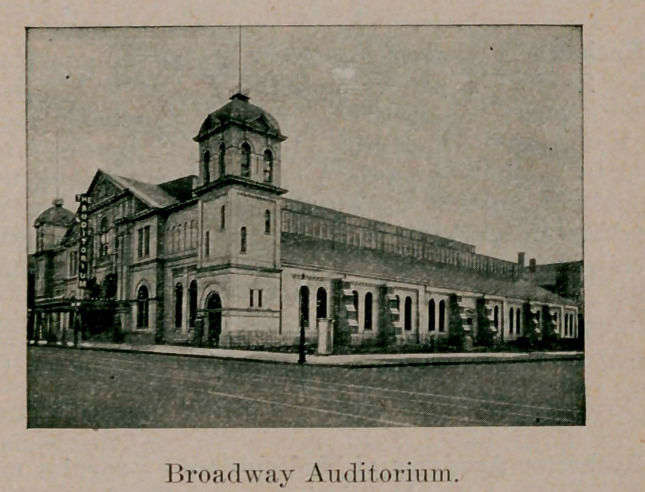# Society Meetings

**Published:** 1915-04

**Authors:** 


### SOCIETY MEETINGS

Brief reports and announcements of meetings of societies of Western New York, and those of general scope, are requested from Secretaries. Copy should be on hand the fifteenth of the month. Full transactions will be published at cost of composition.

**DOCTOR: Is your Society properly represented here? If not, please bring our standing announcement to the attention of the members.**

**Medical Society of the State of New York.** In Buffalo, Tuesday, April 27, to Thursday, April 29. All physicians should attend, whether members of this society or not. The profession is invited from other states and Canada, as well. No special individual railroad rates are granted. Buy regular transportation, unless parties are organized by local railroad agents. As all activities of the meeting, scientific sessions, scientific and commercial exhibits, etc., are held under one roof, in the 65th Regiment Armory (Masten and North to Best streets), and as a restaurant and all other conveniences will be available, arrange for accommodations at hotels or lodging houses accordingly, and come prepared to spend the day, and evening. The exceptions are as follows: The meetings of the

65th Regiment Armory, Largest in the World

Where State Society Will Meet

House of Delegates will be held Monday evening at the Hotel Iroquois. The annual banquet, price $5 will be held Wednesday evening at the Hotel Statler. The New York State Institution for the Study of Malignant Diseases (Gratwick Laboratory, High and Oak Streets about half a mile from the Armory) will be open to visitors at various times, accommodating about 50 in a party. To avoid delay, inquiry for tickets should be made in the Armory. Other special points of interest to medical visitors will be announced by bulletins, etc.

Do not forget the review of the 65th Regiment by Gen. Gorgas, on Friday evening.

Registration, securing of buttons, arrangements for mail, telephones, writing and restrooms, and all similar conveniences will be available in accordance with the usual precedent. Special badges will designate members of the entertainment committee and assistants who will be on hand to furnish information on any point, to give directions and to show guests all possible courtesy. Special arrangements will be made for ladies, including a tea.

The sub-committees whose chairmen constitute the general committee of arrangements for the society, are as follows:

New Marine Bank Building.

RECEPTION: Chas. G. Stockton, chairman, 436 Franklin Street; Arthur W. Hurd, Henry R. Hopkins, William H. Thornton, Henry C. Buswell, Herman E. Hayd, Edward J. Meyer, Harvey P. Gaylord, DeLancey Rochester, Allen A. Jones, Edgar R. McGuire, Thomas J. Walsh, Bernard Cohen, James A. Gardner, Francis E. Fronczak, Lee Masten Francis.

MEETING ROOMS: Nelson G. Russell, chairman, 469 Franklin street; Albert H. Briggs, Renwick R. Ross, Stephen Y. Howell, Theodore M. Leonard, Arthur C. Schaefer.

PUBLICITY: A. L. Benedict, chairman, 228 Summer St.; William W. Quinton, George A. Himmelsbach.

LADIES: Edith R. Hatch, chairman, 2620 Main Street; Maude J. Frye, Myrtle A. Hoag, Lucy A. Kenner, Caroline Lichtenberg, Elizabeth Dort, Katherine Munhall, N. Victoria Chappell, Helene J. C. Kuhlmann, Natalie Mankell.

LOCAL TRANSPORTATION (street cars, cabs, etc.) Dr. D. C. McKenney, 1250 Main Street.

TRANSPORTATION: Carl G. Leo-Wolf, chairman, 481 Franklin Street; William Gaertner, Robert E. DeCeu, Edward M. Tracy, Nelson W. Strohm.

BANQUETS AND HOTELS: Lesser Kauffman, chairman, 534 Elmwood Avenue; Joseph F. Whitwell, William G. Bissell, Earl P. Lothrop, Frederick J. Parmenter.

EXHIBITS AND AUDITS: Albert T. Lytle, chairman, 200 Lexington Avenue, Arthur G. Bennett, Julius Richter.

REGISTRATION AND INFORMATION: Edw. A. Sharp, chairman, 481 Franklin Street; John R. Gray, Clayton M. Brown, John L. Butsch, William L. Phillips, Frank N. Potts, Descum C. McKenney, Herman K. DeGroat, William F. Jacobs, Herbert A. Smith, William Ward Plummer, Augustus W. Hengerer, Nadina R. Kavinoky.

Entrance to Buffalo Harbor.

Taxicab charges will be a dollar a trip between any of the principal hotels and the 65th Armory, for any number of passengers up to 4 for the Miller cabs; up to 5 for the Buffalo taxicabs. For sight-seeing, a rate of $2.50 an hour exists, with the same limits. The street cars are convenient between depots and hotels and strangers should inquire which car to take to avoid transfer. Between the Armory and hotels, there is no convenient street car route without transfer but it is hoped that special cars will be provided to avoid transfer. Strangers should bear in mind that Buffalo has a straight 5-cent car

Tuberculosis Camp, Kensington Avenue fare, tickets being sold without reduction and only in large blocks to accommodate those wishing to keep accurate expense accounts. The fare entitles one to a ride from any point to any other in the city, by a direct route, involving not more than two transfers. Transfers must be punched for the final route. It is probable that sight-seeing autos or other carry-alls will be provided to convey passengers between the hotels and the Armory.

The Committee on Arrangements respectfully requests that all unofficial entertaining be done at times that will not conflict with the regular program of the society.

Members of the profession having photographs of interesting cases are requested to communicate with the chairman, Dr. A. T. Lytle, Lexington and Elmwood avenues, Buffalo, in order that an exhibit may be arranged.

Tuesday evening has been set aside especially for X-Ray exhibits, by co-operation between an informal organization of Buffalo Roentgenologists and commercial firms. This organization will probably be made permanent. Other Roentgenologists are requested to co-operate, addressing Dr. John M.

McKinley Memorial, Niagara Square.

Garratt, 52 North Pearl Street, Buffalo, chairman of the subcommittee on Roentgenology.

The completed program of the meeting will be mailed to all members of the State and County Societies and to others who may specially request it.

As we have already (in the March issue) included some description of Buffalo, we will say nothing further about our

Scene in Delaware Park.

Unloading Ore at Buffalo.

city but will print some illustrations kindly loaned by the Buffalo Chamber of Commerce.

**The American Society for Physicians' Study Travels** will conduct a trip from Philadelphia to San Francisco, starting Sunday, June 8, via Pittsburgh, St. Louis, Denver, Colorado Springs (auto to Crystal Park), Glenwood Springs, Salt Lake City (auto about city, electric road to Saltair Beach), Los Angeles (3 days, side trip to Catalina Islands, ostrich farm, etc.), Santa Barbara (carriage to old mission), Delmonte (auto to Cypress Point), Big Trees, arriving at San Francisco, Sunday, June 20. Leaving Friday, June 25, the return journey will include Portland, Spokane, Lake MacDonald, Glacier Park, Yellowstone Park, St. Paul, Rochester, Minn., Chicago, etc., arriving at Philadelphia, Monday, July 12. The entire price will be $445 or $384 from St. Louis to Chicago. Application should be made promptly to the Sec.-Gen. Dr. Albert Bernheim, 1225 Spruce Street., Philadelphia.

Broadway Auditorium.

**The House of Delegates** of the newly organized **Associated Alumni of the University of Buffalo** met on March 3d and elected the following officers: President, George F. Cott, M. D., '84, president of the Medical Alumni; vice-president, Willis G. Gregory, M. D., '82, Ph. G., '86, dean of the college of pharmacy; secretary, Julian Park, secretary of the Dental Alumni. According to the constitution which was adopted at the dinner on University Day, the new association is to consist of five members, the alumni associations of the departments of medicine, pharmacy, law, dentistry, and analytical chemistry, with the addition of the arts alumni association when it shall be organized. There are three delegates from each department, apportioned as follows: the dean of the department, the president of the alumni association, and a member at large designated by the president. The arts faculty is to designate their member. It is a workable system of organization, and should be productive of a university spirit among the alumni as well as a merely departmental spirit.

---

**The American Hospital Association** will meet in San Francisco, June 22 to 25. Initiation fee including first year's dues $5, subsequent dues $2. The secretary is Dr. H. A. Boyce, Kingston, Canada.

---

**The Medical Society of the County of Monroe** held a regular meeting at the Association Home, 33 Chestnut Street, Rochester, March 16. Dr. W. L. Moss, Internist of the Gratwick Laboratory, Buffalo, spoke on the Treatment of the Anaemias and Haemorrhagic Diseases.

---

**The Rochester Pathological Society** at its regular meeting of February 25, had a paper from Dr. J. F. W. Whitbeck of Rochester, "Brief Comment on Certain Principles in the Practice of Medicine." On March 11, Dr. E. C. Rosenow of Chicago illustrated by stereopticon and gross specimens, the Experimental Production of Appendicitis, Ulcer of Stomach, Cholecystitis and Erythema Nodosum, following Intravenous Injection of Streptococci from the Corresponding Diseases in Man.

March 25, Dr. A. D. Stewart read a paper on "Causation and Treatment of Leucorrhoea." The officers are: C. F. Chaffe, president; H. J. Vary, secretary.

---

**The Rochester Academy of Medicine** held a regular meeting with Section III at the Association rooms, 33 Chestnut Street, Wednesday, March 10, 1915, at 8.30 p. m. Topics: Addison's Disease, Dr. George A. Marion; The Therapeutic Use of Epiniphrin in Rhinolaryngology, Dr. N. D. McDowell; The Therapeutic Use of Epinephrin in Ophthalmology, Dr. L. W. Jones; The Therapeutic Use of Epinephrin in Surgical Shock, Dr. W. B. Jones.

---

**The Buffalo Academy of Medicine** has held the following meetings since last noted: Section of Pathology, February 24, Experimental Studies of Spleen in Relation to Anaemia, Haemolysis and Haemolytic Jaundice, Dr. R. M. Pearce of Philadelphia, discussion opened by Dr. Irving P. Lyon. Section of Surgery, March 3, Early Symptoms of Intussesception, Drs. Irving M. Snow and Fred Parmenter; Gun-shot Wounds in the Present War, Dr. David Wheeler (Read by Major W. W. Quinton, Dr. Wheeler being absent in the Red Cross Service.) March 6, under the auspices of the Academy, Dr. Richard C. Cabot of Boston gave a clinic at the Buffalo General Hospital. Section of Medicine, March 10, Eternal Verities in Tuberculosis, Dr. E. S. Bullock, New Mexico Cottage Sanitarium; Gynaecology, from the Medical Men's Viewpoint, Dr. Ray H. Johnson. Section of Obstetrics and Gynaecology, March 17, providing program for Stated Meeting of Academy. Mental Disorders of Pregnancy and the puerperium, Dr. Herman G. Matzinger; The Healing Art in the Philippines under Uncle Sam, (stereopticon views) Dr. Stephen Yates Howell. Nominations for officers for the coming year were made as follows: for president, Drs. Lawrence Hendee and James Wright Putnam; for treasurer, Dr. Theodore M. Leonard; for Secretary, the incumbent, Dr. Herbert A. Smith.

---

**The Hospital Medical Society of Rochester,** held its regular meeting March 18. Dr. A. B. Kaiser read a paper on Schick's Reaction.

---

**Women's Medical Society of New York State**—The ninth annual meeting will be held at Hotel Statler, Buffalo, N. Y., Monday, April 26, 1915. Meeting called, 9:30 a. m.; banquet, 7:30 p. m. All women physicians cordially invited. Program:

Invocation—Eliza M. Mosher, M. D., Brooklyn, N. Y.

Address of welcome—Helena J. C. Kuhlman, M. D., Buffalo, N. Y.

Greetings from the Medical Society of New York State—President Grover W. Wende, M. D., Buffalo, N. Y.

Response—Dr. Grace Peckham Murray.

President's address—Evolution and Revolution—25 years of Obstetrics in retrospect—Angenette Parry, M. D., New York City.

Internal Secretions in the Treatment of Sterility—Caroline S. Finley, M. D., New York City.

Hypothyroidism with report of Case—M. Louise Hurrell, M. D., Rochester, N. Y.

Hyperplasia of the Endometrium—Elizabeth Hurdon, M. D., C. M., Baltimore, Md.

Afternoon:

Notes on a British Spa—Eleanor Parry, M. D., New York City.

The Province of Development and Corrective Exercise as illustrated by the method of W. Curtis Adams (fifth demonstration)—Stella S. Bradford, M. D., Montclair, N. J.

The Modern Method in Obstetrics—Bertha Van Hoesen, A. M., M. D., Chicago.

The Significance of the Wasserman Reaction from a Laboratory Standpoint—Elise S. L'Esperance, M. D., New York City.

Report of a case of pelvic injury—Harriet M. Doane, M. D., Fulton, N. Y.

A Plea for Correct Diagnosis of Pelvic Lesions—N. Victoria Chappell, M. D., Buffalo, N. Y.

The officers of the Women's Medical Society of New York State for 1915, are as follows:

President, Angenette Parry, M. D., 749 Madison Avenue, New York City.

First Vice-president, Cort Billings Lattin, M. D., Buffalo.

Second Vice-president, Evelyn Baldwin, M. D., 476 West Avenue, Rochester.

Third Vice-president, Mathilda Wallin, M. D., 616 Madison Avenue, New York City.

Secretary, Edith R. Hatch, M. D., 2620 Main Street, Buffalo.

Treasurer, Cornelia White-Thomas, M. D., 470 Lyell Avenue, Rochester.

Councilors—1st Dist., Eleanor Parry, M. D., 749 Madison Avenue, N. Y. City; 2d Dist., Anna Craig, M. D., King's Park, L. I.; 3d Dist., Julia G. McNutt, M. D., 126 State Street, Albany; 4th Dist., Maria H. Hopkins, M. D., Gloversville; 5th Dist., Angeline Martine, M. D., Utica; 6th Dist., Esther M. Parker, M. D., 326 E. State Street, Ithaca; 7th Dist., Eveline P. Ballintine, M. D., Rochester State Hospital, Rochester; 8th Dist., Maud J. Frye, M. D., 211 Highland Avenue, Buffalo.

Scientific Program—Kathleen Buck, M. D., Rochester, N. Y.; E. Winifred Pitkin, Congers, N. Y.

Legislation—Marie Chard, M. D., 616 Madison Avenue, New York City; Mary Green, M. D., Castile; Edith Stewart, M. D., Hume.

Public Health—Phoebe M. VanVoast, M. D., 352 East 200th Street, New York City; Esther L. Jefferis, M. D., 109 East 56th Street, New York City; M. Louise Hurrell, M. D., 1657 East Avenue, Rochester.

Arrangements—Sarah G. Pierson, M. D., R. S. H., Rochester; Nathalie K. Mankell, M. D., Buffalo; Florence McKay, M. D., Lake Avenue, Rochester.

Toast Mistress—Rosalie Slaughter Morton, M. D., 709 Madison Avenue, New York City.

## Figures and Tables

**Figure f1:**
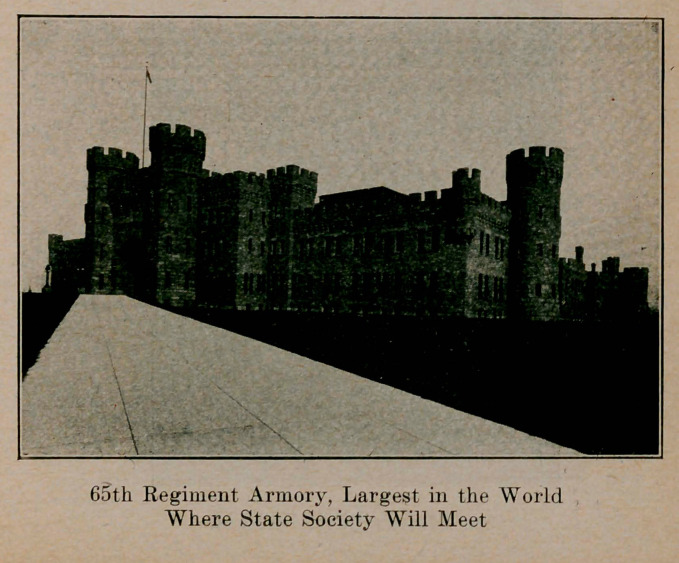


**Figure f2:**
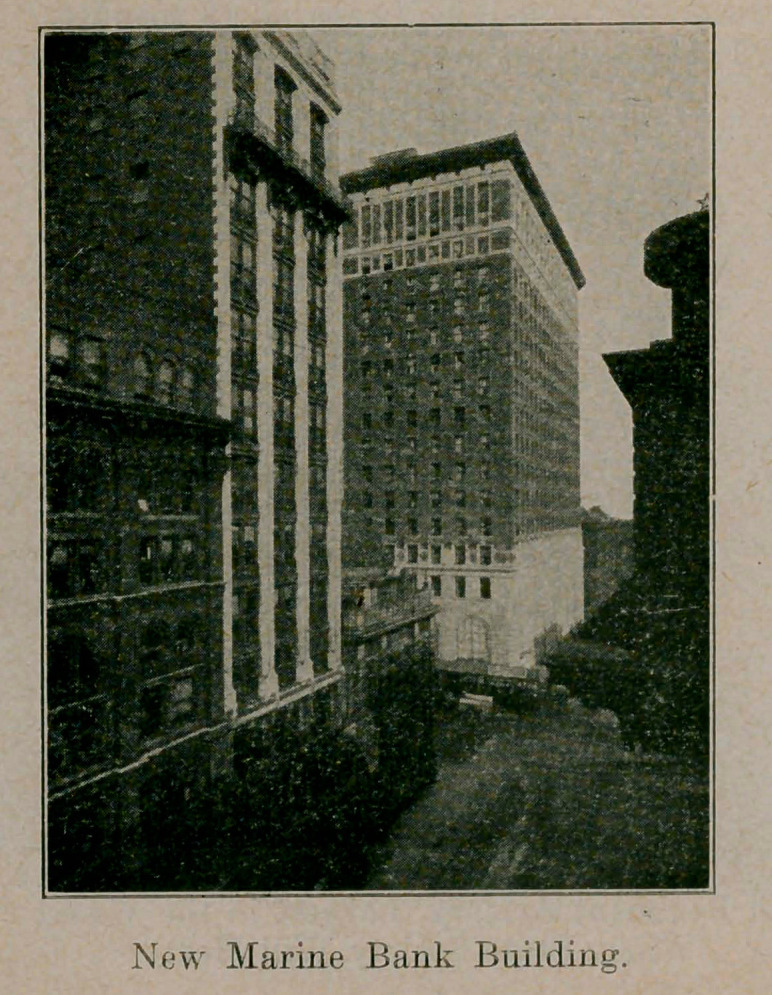


**Figure f3:**
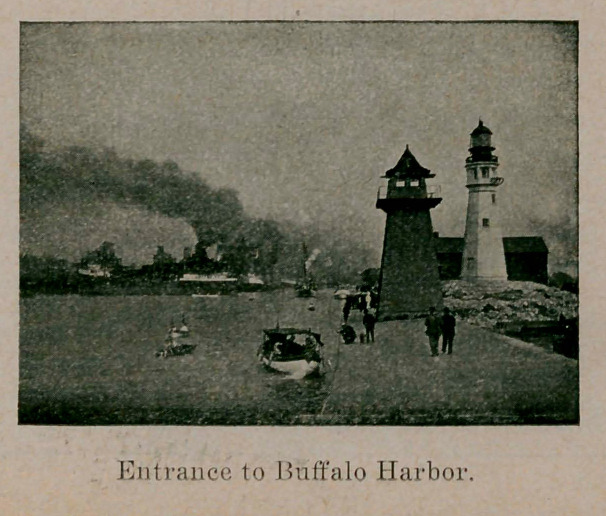


**Figure f4:**
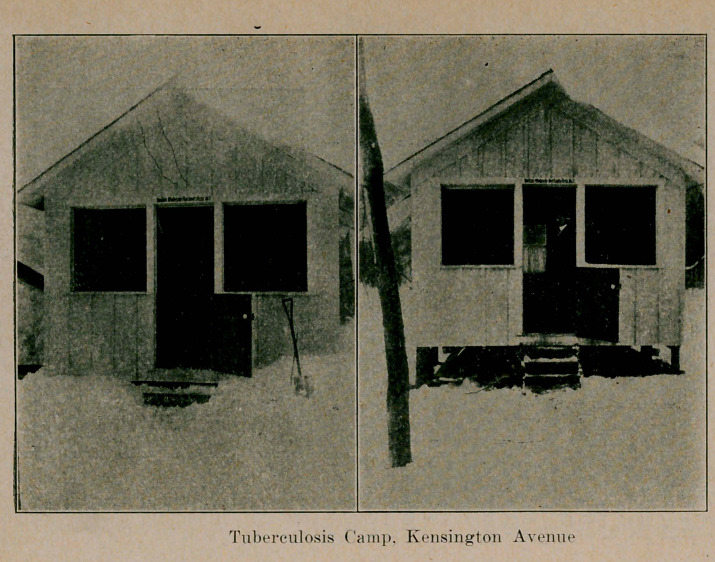


**Figure f5:**
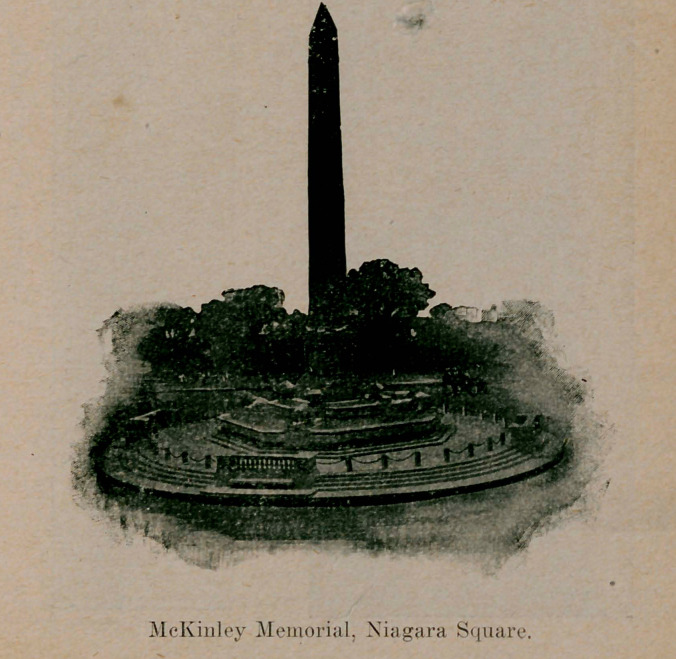


**Figure f6:**
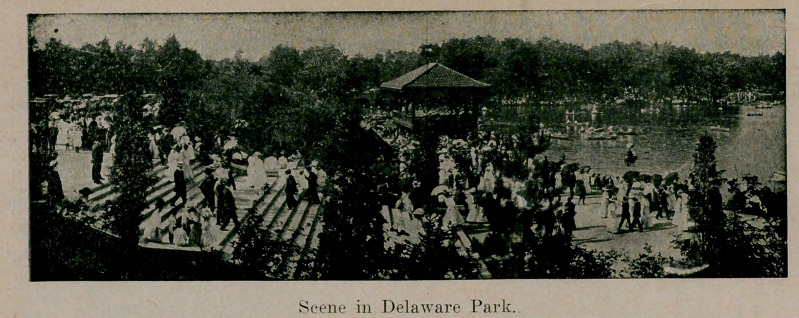


**Figure f7:**
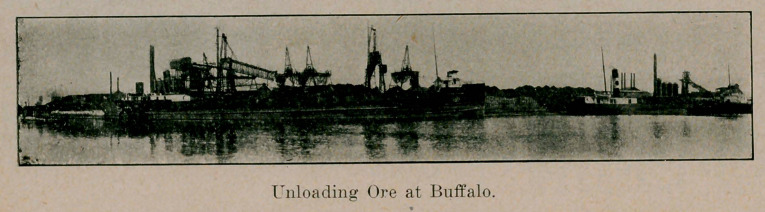


**Figure f8:**